# Supplementary material for: Resolving the full spectrum of human genome variation using Linked-Reads
Source: Genome Res. 2019 Apr;29(4):635–45. doi: 10.1101/gr.234443.118 (PMC6442396; doi:10.1101/gr.234443.118)
Supplement: Supplemental Material [file supp_gr.234443.118_Supplemental_longranger-2.2.zip › longranger-2.2.tar.bz2!/lr2.2-public/lib/stan/cmdstan/stan/doc/api/html/math_2matrix_2mdivide__left__ldlt_8hpp_source.html]

Stan: src/stan/math/matrix/mdivide\_left\_ldlt.hpp Source File


|  |  |
| --- | --- |
|  | Stan  2.5.0  probability, sampling & optimization |


- Main Page
- Related Pages
- Namespaces
- Classes
- Files

- File List
- File Members

All Classes Namespaces Files Functions Variables Typedefs Enumerations Enumerator Friends Macros Pages

- src
- stan
- math
- matrix

mdivide\_left\_ldlt.hpp

Go to the documentation of this file.

1 #ifndef STAN\_\_MATH\_\_MATRIX\_\_MDIVIDE\_LEFT\_LDLT\_HPP

2 #define STAN\_\_MATH\_\_MATRIX\_\_MDIVIDE\_LEFT\_LDLT\_HPP

3

4 #include <boost/math/tools/promotion.hpp>

5 #include <stan/math/matrix/Eigen.hpp>

6 #include <stan/math/matrix/LDLT\_factor.hpp>

7 #include <stan/math/error\_handling/matrix/check\_multiplicable.hpp>

8 #include <stan/math/matrix/promote\_common.hpp>

9 #include <boost/type\_traits/is\_same.hpp>

10

11 namespace stan {

12  namespace math {

13

22  template <int R1,int C1,int R2,int C2, typename T1, typename T2>

23  inline Eigen::Matrix<typename boost::math::tools::promote\_args<T1,T2>::type,R1,C2>

24  mdivide\_left\_ldlt(const stan::math::LDLT\_factor<T1,R1,C1> &A,

25  const Eigen::Matrix<T2,R2,C2> &b) {

26  stan::math::check\_multiplicable("mdivide\_left\_ldlt(%1%)",A,"A",

27  b,"b",(double\*)0);

28

29  return A.solve(promote\_common<Eigen::Matrix<T1,R2,C2>,

30  Eigen::Matrix<T2,R2,C2> >(b));

31  }

32

33  }

34 }

35 #endif

check\_multiplicable.hpp

promote\_common.hpp

stan::math::mdivide\_left\_ldlt

Eigen::Matrix< typename boost::math::tools::promote\_args< T1, T2 >::type, R1, C2 > mdivide\_left\_ldlt(const stan::math::LDLT\_factor< T1, R1, C1 > &A, const Eigen::Matrix< T2, R2, C2 > &b)

Returns the solution of the system Ax=b given an LDLT\_factor of A.

**Definition:** mdivide\_left\_ldlt.hpp:24

stan::math::check\_multiplicable

bool check\_multiplicable(const char \*function, const T1 &y1, const char \*name1, const T2 &y2, const char \*name2, T\_result \*result)

**Definition:** check\_multiplicable.hpp:15

stan::math::LDLT\_factor

**Definition:** LDLT\_factor.hpp:16

Eigen.hpp

stan::math::promote\_common

common\_type< T1, T2 >::type promote\_common(const F &u)

**Definition:** promote\_common.hpp:14

LDLT\_factor.hpp


---

[ Stan Home Page ]

*© 2011–2014,
Stan Development Team.*
